# Recommendations on the surveillance and supplementation of vitamins and minerals for upper gastrointestinal cancer survivors: a scoping review

**DOI:** 10.1007/s11764-024-01666-4

**Published:** 2024-08-29

**Authors:** Sim Yee (Cindy) Tan, Tiffany Tsoukalas, Kirsten Javier, Tiffany Fazon, Sheena Singh, Janette Vardy

**Affiliations:** 1 Sydney Medical School, University of Sydney, Concord, NSW Australia; 2 Concord Cancer Centre, Concord Hospital, Concord, NSW Australia; 3 Nutrition and Dietetics Department, Concord Hospital, Concord, NSW Australia; 4 Discipline of Nutrition and Dietetics, Sydney Nursing School, Faculty of Medicine and Health, University of Sydney, Camperdown, Australia; 5 Cowra Community Health, Cowra Health Service, Cowra, NSW Australia; 6 Psycho-Oncology Cooperative Research Group (PoCOG), School of Psychology, Faculty of Science, University of Sydney, Camperdown, NSW Australia

**Keywords:** Upper gastrointestinal cancer, Survivorship care, Micronutrient deficiencies, Mineral and vitamin supplements, Cancer survivors

## Abstract

**Background:**

Early-stage upper gastrointestinal (UGI) cancer patients, after surgery, have altered gastrointestinal functions, compromising their nutritional status and health outcomes. Nutritional care provision to UGI survivors rarely focuses on long-term survivorship. Here, we explore recommendations for surveillance of micronutrient deficiency and supplementation for UGI cancer survivors after surgery.

**Methods:**

A scoping review, based on the Joanna Briggs Institute methodology for scoping reviews. Six databases (Medline, Embase, CINAHL, Cochrane, Scopus, and PsycINFO) and 21 cancer-related organisation websites were searched. Publications between 2010 and March 2024 with recommendations aimed at adult UGI cancer (oesophageal, gastric, pancreatic, small bowel, and biliary tract) survivors were included.

**Results:**

Twenty-six publications met the selection criteria: 11 reviews (8 narrative reviews, 2 systematic, 1 meta-analysis), 7 expert opinions, 6 guidelines, and 2 consensus papers. Twenty-two publications recommended monitoring of micronutrient deficiencies, and 23 suggested supplementation, with 8 lacking details. Most were targeted at patients with gastric cancer (*n* = 19), followed by pancreatic cancer (*n* = 7) and oesophageal cancer (*n* = 3) with none for biliary tract and small bowel cancers. Vitamin B12 and iron were the most consistently recommended micronutrients across the three tumour groups.

**Conclusion:**

Limited publications recommend surveillance of micronutrient status in UGI cancer survivors during the survivorship phase, especially for oesophageal and pancreatic cancer survivors; most were narrative reviews. These recommendations lacked details, and information was inconsistent.

**Implications for cancer survivors:**

Long-term UGI cancer survivors are at risk of micronutrient deficiency after surgery. A standardised approach to prevent, monitor, and treat micronutrient deficiencies is needed.

**Supplementary Information:**

The online version contains supplementary material available at 10.1007/s11764-024-01666-4.

## Introduction

In recent years, the 5-year survival rate of patients with early-stage upper gastrointestinal (UGI) cancer has improved. In Australia, gastric and pancreatic cancer 5-year relative survival rates improved from 21 to 38% and from 3.7 to 13%, between 1990 and 1994 and between 2015 and 2019, respectively [1, 2]. Similarly, between 1989 and 1993 and between 2014 and 2018, there was a 9% improvement (from 14 to 23%) in the 5-year relative survival for oesophageal cancer [3]. Whilst this improvement in patient survival is reflective of current medical advancements in screening and treatment, UGI cancer patients frequently experience long-term complications due to their treatment [4, 5].

Nutritional deficiencies are a common sequela associated with the anatomical and functional modification of a patient’s gastrointestinal tract post oesophagectomy, gastrectomy, pancreatectomy, and/or Whipple’s procedure [5–9]. A recent systematic review with meta-analysis of 2627 patients after gastrectomy for gastric cancer found almost 1 in 2 patients (48.8%) had B12 deficiencies after a total gastrectomy [7]. Similarly, a recent retrospective cohort study found the incidence of anaemia (caused by either iron or B12 deficiency) steadily increased 5 years post gastrectomy [10]. Janssen et al. [11] reported 78% of patients 6 months after oesophagectomy had at least one micronutrient deficiency. This rate remained high at a median follow-up of 24.8 months, although the number of patients remaining in the follow-up cohort was small [11].

To improve survivors’ quality of life (QoL) and overall health, clinicians need to recognise and manage the symptoms and side effects associated with cancer treatments [12, 13]. At present, there is no standard practice for long-term nutritional care for UGI cancer survivors post-surgical resection. Many of the nutrition-related guidelines [14, 15] for cancer survivors either are not UGI cancer-specific, lack focus on the long-term survivorship phase [16, 17], or do not specify if monitoring of micronutrient status is needed for UGI cancer survivors [18, 19]. Therefore, the aim of this study is to explore the literature on the recommendations for long-term surveillance and supplementation of vitamins and minerals in UGI cancer survivors after curative intent surgery.

## Methods

A protocol was developed according to the Joanna Briggs Institute methodology guideline for scoping reviews; the PRISMA extension for scoping reviews (PRISMA-ScR) was used to guide the reporting [20, 21].

### Eligibility criteria

The ‘Population, Intervention, Comparator, Outcomes, Study Design’ (PICOS) framework was used to guide the development of the selection criteria [22]. The target population was UGI cancer survivors, of adult age (18 and over). For the purpose of this review, UGI cancer survivors refer to individuals who have been diagnosed with either oesophageal, gastric, pancreatic, biliary, or small intestine cancer; have completed treatment with curative intent; and remain cancer free. Curative intent UGI surgeries included oesophagectomy, gastrectomy, pancreatectomy, cholecystectomy, and Whipple’s procedure, for TNM stage T1-3, N0-2, and M0 cancers. Outcomes of interest included guidelines, consensus statements, literature reviews, and/or white paper/position statements (endorsed by a reputable organisation) which contained recommendations for the surveillance of micronutrient status and supplementation during the survivorship phase for the study population. The survivorship phase here refers to the time after curative intent treatment, until the time of cancer recurrence or death (whichever comes first). Publications from randomised controlled trials, cohort studies, retrospective audit, or case studies were excluded. Publications must have been published between 2010 and 2024, with the full text available in the English language.

### Sources of information

Six databases (Medline, Embase, CINAHL, Cochrane, Scopus, and PsycINFO) were searched in September 2021 for relevant articles, with updated searches in February 2023 and March 2024. In addition to the databases, 21 websites (15 professional organisations (European Society for Clinical Nutrition and Metabolism (ESPEN), American Society for Parental and Enteral Nutrition (ASPEN), Australasian Society for Parental and Enteral Nutrition (AuSPEN), European Society for Medical Oncology (ESMO), American Society of Clinical Oncology (ASCO), Clinical Oncology Society of Australia (COSA), Practice-Based Evidence in Nutrition, British Society of Gastroenterology, National Institute for Health and Care Excellence (NICE), International Study Group on Pancreatic Surgery (ISGPS), International Society of Geriatric Oncology (SIOG), European Society of Gastrointestinal Endoscopy (ESGE), Pancreatic Cancer Action Network (PanCAN), National Comprehensive Cancer Network (NCCN), UpToDate); four cancer organisations (European Organisation for the Research and Treatment of Cancer (EORTC), World Cancer Research Fund (WCRF), Cancer Council Australia, American Cancer Society (ACS)); and two government sources (Cancer Australia, National Cancer Institute)) were searched for relevant publications (see Supplementary Table 2).

### Search strategy

The search strategy was developed in consultation with an academic librarian from the University of Sydney. A combination of search terms relating to ‘upper gastrointestinal cancer’, ‘surgery’, ‘cancer survivor or patient’, ‘outpatient or post discharge’, ‘guideline or recommendation’, and ‘vitamin and mineral supplement’ was used to search the six databases (see Appendix 1 for the search strategy for Medline).

### Selection process

EndNote 20, a reference managing software, was used during the selection process to import the searches from the databases; and Covidence, an online systematic review tool, was used to remove duplicates, and for screening (of both abstract and title and full text). Grey literature and publications obtained from the websites/hand searches were imported into Covidence directly. Each of the records was screened by two independent reviewers (KJ and SYT at the initial search, TF and SYT and TT and SYT for the updated searches). Publications were excluded if they were duplicates or did not meet the inclusion criteria. Literature reviews with no clear statement regarding recommendations for surveillance of any micronutrient deficiency and/or supplementation were excluded. Full-text publications that did not meet the inclusion criteria were excluded based on a pre-established order of exclusion list, to ensure consistency between the reviewers. Any discrepancies between the reviewers at the screening stage were resolved by consensus first, and a third reviewer was used (SS or JV) if consensus was unable to be reached (Table 1).

**Table 1:** General characteristics of the included publications (*n* = 26)

Study characteristics	*N* (%)
Publication year	
2010–2019	8 (31)
2020–2024	18 (69)
Study design	
Literature reviews	11 (42)
Narrative	8 (31)
Systematic	2 (8)
Systematic with meta-analysis	1 (4)
Expert opinions	7 (27)
Guidelines	6 (23)
Consensus statements	2 (8)
Source	
Manuscript publications	17 (65)
Book chapters	3 (12)
Website — National Comprehensive Cancer Network	2 (8)
UpToDate	4 (15)
Country	
USA	12 (46)
Korea	2 (8)
Canada	2 (8)
UK	1 (4)
Italy	1 (4)
Taiwan	1 (4)
Germany	1 (4)
France	1 (4)
Japan	1 (4)
> 1 country	4 (15)
Tumour type	
Gastric	16 (62)
Pancreatic	6 (23)
Oesophageal	1 (4)
> 1 tumour type	3 (12)
Oesophageal and gastric	2 (8)
Gastric and pancreatic	1 (4)

Percentage is reported to the nearest whole number

### Data charting and synthesis of results

For each included publication, the author, year, country (where the authors were based), publication type, intended population (including cancer type and surgery type), and recommendations for micronutrient surveillance and/or supplementation were extracted by two reviewers independently (KJ and SYT at the initial search, TF and SYT, and TT and SYT for the updated searches). Extracted data were recorded on an Excel spreadsheet. The first approach to any conflicts between the reviewers was through discussion between pairs. If consensus was unable to be reached, another team member (JV) was used. For publications with multiple updates, only the latest version was used. The types of publications were categorised as per the title of the publication unless there was documentation about consensus reached among the experts. As an example, NCCN clinical practice guidelines underwent a consensus process among the group of experts, which hence were categorised as a guideline which reached consensus (Table 2). Reviews from UpToDate, an online platform with an established editorial process outlined on the website, and book chapters were classified as ‘expert opinion’. Literature reviews with documented search strategies were classified as systematic reviews. Those without a search strategy were classified as narrative reviews.

**Table 2:** List of published practice guidelines and consensus included recommendations (*n* = 8)

First author, year of publication	Title	Country	Reached consensus	Authors	Study methods/designs	Endorsed by	Conflict of interest declared
Oesophageal cancer (***n*** = 1)							
2003National Comprehensive Cancer Network (NCCN) 2024 [25]	NCCN Clinical Practice Guidelines in Oncology for Esophageal and Esophagogastric Junction Cancers (Version 1.2024)	USA	Yes	Group of experts	Detailed description regarding process of developing the guidelines and consensus (NCCN Categories of Evidence and Consensus)	The National Comprehensive Cancer Network	Yes
Gastric cancer (***n*** = 7)							
DeManzoni 2017 [28]	The Italian Research Group for Gastric Cancer (GIRCG) guidelines for gastric cancer staging and treatment: 2015	Italy	Web-based and Delphi method-based Consensus Conference	Group of experts	No detailed descriptions regarding how consensus was reached and if any grading of evidence was conducted	The Italian Research Group for Gastric Cancer (GIRCG)	None
Hsu 2019 [31]	Taiwan nutritional consensus on the nutrition management for gastric cancer patients undergoing gastrectomy	Taiwan	Yes	Group of experts	Described literature search and process of achieving agreement. Used modified grading system of the Oxford Centre for Evidence-based Medicine Levels of Evidence (March 2009)	Gastroenterological Society of Taiwan	None
Kim 2023 [29]	Korean Practice Guidelines for Gastric Cancer 2022: An Evidence-based, Multidisciplinary Approach	Korea	Yes	Group of experts	Detailed methods of literature review, grading of evidence included	Korean Gastric Cancer Association	Yes
Lordick 2022 [23]	Gastric cancer: ESMO Clinical Practice Guideline for diagnosis, treatment and follow-up	European	Not mentioned	Group of experts	No detailed methods documented but a standard process of literature review and using levels of evidence and grades of recommendation (adapted from the Infectious Diseases Society of America-United States Public Health Service Grading Systema) outlined	European Society for Medical Oncology	Yes
Mulder 2016 [39]	Report from the 17th Annual Western Canadian Gastrointestinal Cancer Consensus Conference; Edmonton, Alberta; 11–12 September 2015	Canada	Yes	Group of experts	Western Canadian Gastrointestinal Cancer Consensus Conference; no further details regarding grading of evidence		Yes
National Comprehensive Cancer Network 2024 [30]	NCCN Clinical Practice Guidelines in Oncology for Gastric Cancer (Version 1.2024—7/3/2024)	USA	Yes	Group of experts	Detailed description regarding process of developing the guidelines and consensus (NCCN Categories of Evidence and Consensus)	The National Comprehensive Cancer Network	Yes
Shitara 2024 [24]	Pan-Asian adapted ESMO Clinical Practice Guidelines for the diagnosis, treatment, and follow-up of patients with gastric cancer	European and Asian	Yes	Group of experts	No detailed methods documented but a standard process of literature review and using levels of evidence and grades of recommendation (adapted from the Infectious Diseases Society of America-United States Public Health Service Grading Systema) have been outlined	European Society for Medical Oncology and Oncological societies of China (CSCO), Indonesia (ISHMO), India (ISMPO), Japan (JSMO), Korea (KSMO), Malaysia (MOS), the Philippines (PSMO), Singapore (SSO), Taiwan (TOS), and Thailand (TSCO), and Japanese Society of Medical Oncology (JSMO)	Yes

Due to the nature of this scoping review, a critical appraisal of the included studies was not carried out.

Descriptive statistics were used to describe the characteristics of the publications and if they contained recommendation(s) for the use of vitamin and/or mineral supplementation and monitoring of micronutrient status in UGI cancer survivors. Details of these recommendations were summarised qualitatively and tabulated.

## Results

### Search results

**Fig. 1:**
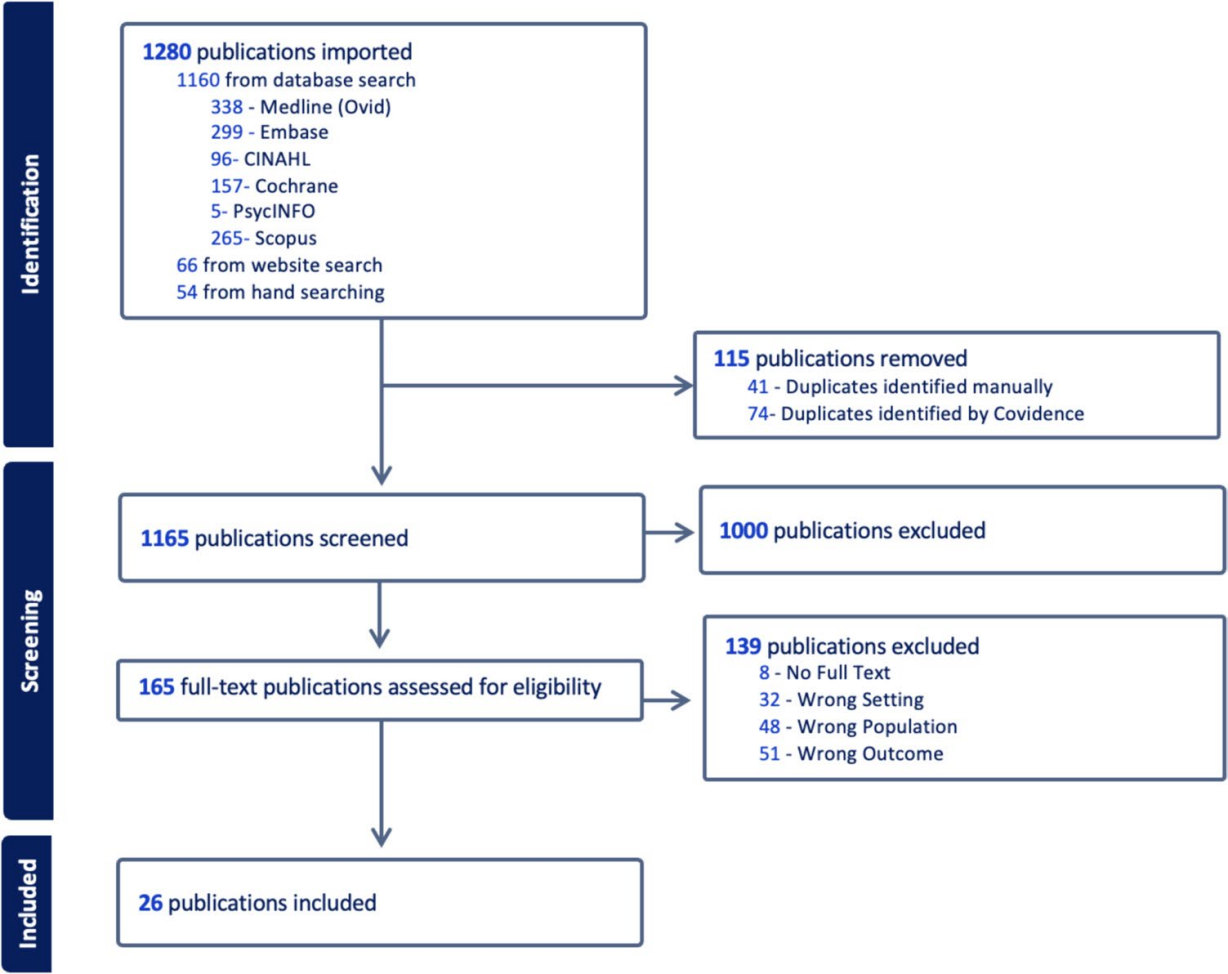
PRISMA flow diagram

The search strategy identified 1280 records: 338 from Medline, 299 from Embase, 96 from CINAHL, 157 from Cochrane, 5 from PsycINFO, and 265 from Scopus. An additional 66 publications were identified from the websites described earlier, and 54 by hand searching (Fig. 1). Overall, 115 records were removed as duplicates; 1165 underwent title and abstract screening, and 165 records underwent full-text review, with 8 records unable to be retrieved for review. There were 131 articles excluded due to the following reasons: wrong setting (*n* = 32); wrong population (*n* = 48); wrong outcomes (*n* = 51) where 30 publications did not have nutrition-related recommendations for cancer survivors and 21 did not have micronutrient-specific recommendations for UGI cancer survivors. In total, 26 articles met the selection criteria for data extraction.

### Characteristics of publications

A total of 26 publications related to oesophageal, gastric, and pancreatic cancer survivors were identified through this scoping review (see Table 1 for the summary). There were no publications related to biliary tract or small intestine cancers. Thus, the remaining scoping review will focus only on the three tumour types mentioned above. Of the 26 publications included, 11 (42%) were literature reviews (8 narrative, 2 systematic, and 1 meta-analysis), 7 (27%) expert opinions, 6 (23%) practice guidelines (with five documented as reaching consensus), and 2 (8%) consensus statements. Most publications (*n* = 16, 62%) were targeted at patients with gastric cancer, followed by pancreatic cancer (*n* = 6, 23%) and oesophageal cancer (*n* = 1, 4%). Three publications included more than one tumour type — two oesophageal and gastric, and one gastric and pancreatic. Most studies were published in the USA (*n* = 12, 46%), followed by European countries (*n* = 4, 15%), Asian countries (*n* = 4, 15%), and Canada (*n* = 2, 8%). The remaining guidelines were issued either by organisations consisting of topic experts from different countries (*n* = 2, 8%) [23, 24] or by researchers from multiple countries (*n* = 2, 8%). Most of the publications (*n* = 18; 69%) were published in the last 5 years.

### Oesophageal cancer

Three publications were relevant to oesophageal cancer survivors post oesophagectomy: 1 guideline [25], 1 meta-analysis [26], and 1 systematic review [27]. All three discussed micronutrient-related deficiencies and suggested surveillance, and two [26, 27] recommended supplementation (see Table 3 for details). The type of vitamins and minerals suggested to be monitored in these three publications varied. NCCN Guidelines for Oesophageal and Oesophagogastric Junction Cancers 2024, a guideline consisting of opinions from a group of experts based on available evidence, recommended monitoring calcium, folic acid, B vitamins, and vitamin D status only [25]. A meta-analysis investigating malnutrition and vitamin deficiencies after surgery for oesophageal and gastric cancer recommended monitoring for vitamin deficiencies and suggested supplementation of vitamin B12, vitamin D, and calcium (for patients with increased risk of osteoporosis, calcium or vitamin D deficiency) [26]. They suggested these patients receive the same vitamin supplement regimen as patients who underwent a sleeve gastrectomy. A literature review that provided an overview of nutrition-related issues post oesophagectomy recommended surveillance of serum vitamin levels (at specific time intervals) and supplementation in case of deficiency [27].

**Table 3:** List of recommendations for micronutrient surveillance and supplementation in cancer survivors post curative intent treatment for upper gastrointestinal tract cancer (*n* = 26)

First author, year of publication (country)	Title (publication type)	Target population	Includes recommendations for surveillance of micronutrient status? (List of micronutrients specified in text if available)	Includes recommendations for micronutrient supplementation? (List of micronutrients specified in text if available)
Oesophageal cancer (***n*** = 3)				
Finze et al., 2024 (Germany and Netherlands) [26]	Malnutrition and vitamin deficiencies after surgery for esophageal and gastric cancer: A metanalysis (Meta-analysis)	Oesophageal and gastric cancer patients	Yes — monitor for vitamin deficiencies post oesophagectomy	Yes - Vitamin B12 and D post oesophagectomy - Calcium (for patients with increased risk of osteoporosis, calcium deficiency, or high risk of vitamin D deficiency) - Recommend vitamin supplement regimen similar to patients having sleeve gastrectomy
National Comprehensive Cancer Network^a^, 2024 (NCCN) (USA) [25]	NCCN Clinical Practice Guidelines in Oncology for Esophageal and Esophagogastric Junction Cancers (Version 1.2024) (Guideline — reached consensus)	Oesophageal cancer patients	Yes — calcium, folic acid, vitamin B (non-specific), vitamin D	No
Teixeira Farinha et al.^a^, 2023 (France and Switzerland) [27]	Gastro-Intestinal Disorders and Micronutrient Deficiencies (Systematic review)	Oesophageal and gastric cancer patients	Yes — monitor vitamin levels (post oesophagectomy)	Yes — in case of deficiency
Gastric cancer (***n*** = 19)				
Cobani et al.^a,b^, 2023 (USA) [32]	Gastric Cancer Survivorship: Multidisciplinary Management, Best Practices and Opportunities (Narrative review)	Gastric cancer survivors	Yes: - B12, iron (if deficient) - Bone mineral density	Yes - B12 (as per clinical indications) - Iron (if deficient; post total gastrectomy: avoid enteric coated or sustained release formulations) - Vitamin D (if at risk for osteoporosis)
DeManzoni^a^ et al., 2016 (Italy) [28]	The Italian Research Group for Gastric Cancer (GIRCG) guidelines for gastric cancer staging and treatment 2015 (Guideline — reached consensus)	Gastric cancer patients	Yes — including but not limited to vitamin B12, iron, and calcium	Yes — including but not limited to vitamin B12, iron, calcium
Finze et al., 2024 (Germany and Netherlands) [26]	Malnutrition and vitamin deficiencies after surgery for esophageal and gastric cancer: A metanalysis (Systematic review and meta-analysis)	Oesophageal and gastric cancer patients	Yes — monitor for vitamin deficiencies post gastrectomy	Yes - Vitamin B12 and D (post gastrectomy) - Calcium (for patients with increased risk of osteoporosis or calcium deficiency, or high risk of vitamin D deficiency) - Recommended vitamin supplement regimen similar to patients who have undergone Roux-en-Y gastric bypass surgery
Hebbard et al., 2024 (Canada) [38]	Partial gastrectomy and gastrointestinal reconstruction (Expert opinion — UpToDate)	Gastric cancer patients post partial gastrectomy and gastrointestinal reconstruction	No	Yes - Vitamin B12 post subtotal gastrectomy - Iron and calcium post gastrectomy - Fat-soluble vitamins (may be required post reconstructive surgery that bypasses the duodenum (e.g. Roux-en-Y, Billroth II))
Hsu et al.^a,b^, 2019 (Taiwan) [31]	Taiwan nutritional consensus on the nutrition management for gastric cancer patients receiving gastrectomy (Consensus statement)	Gastric cancer patients, after gastrectomy	Yes - B12 (only post subtotal gastrectomy) - Iron (serum ferritin and haemoglobin), 25-hydroxy vitamin D, zinc, and copper - Bone mineral density	Yes - Prophylactic daily multivitamin supplement, B12 - Iron, folate (if deficient) - Zinc and copper (when clinically indicated) - Calcium and vitamin D (in case of low bone mineral density)
Jacobson et al., 2023 (USA) [5]	Long‐term nutrition alterations after surgery for gastrointestinal cancers (Narrative review)	Patient with gastrointestinal cancers (including colorectal) after surgery (including Whipple procedure, gastrojejunostomies, and/or total gastrectomy)	Yes — vitamin B12 (post total gastrectomy)	Yes - Vitamin B12, iron, and folate (post total gastrectomy) - Vitamin C (optional; to enhance absorption of iron)
Kim et al.^a^, 2023 (Korea) [33]	Advances, breakthroughs, and challenges in gastric cancer surgery (Narrative review)	Patients after gastric surgery	Yes — particularly iron and vitamin B12	No
Kim^b^ et al., 2023 (Korea) [29]	Korean Practice Guidelines for Gastric Cancer 2022: An Evidence-based, Multidisciplinary Approach (Guideline — reached consensus)	Gastric cancer patients	Yes — B12	Yes - B12 - Iron (if deficient) - Vitamin D and calcium (if with increased osteoporosis risk)
Lordick et al., 2022 (European) [23]	Gastric cancer: ESMO Clinical Practice Guideline for diagnosis, treatment and follow-up (Clinical practice guideline)	Gastric cancer patients	Yes — no details	No
Malik et al.^a,b^, 2020 (USA) [34]	Nutritional Implications in Preparing Patients for Total Gastrectomy (Narrative review)	Patients post total gastrectomy	Yes - Fat-soluble vitamins, iron, calcium, and folate - Bone mineral density	Yes - Vitamin B12, multivitamin (containing all minerals and vitamins) - Folate, iron, fat-soluble vitamins (if deficient) - Calcium (patients with known bone disease)
Mamon et al., 2024 (USA) [37]	Adjuvant and neoadjuvant treatment of gastric cancer (Expert opinion — UpToDate)	Gastric cancer patients	Yes — vitamin B12 and iron (particularly post total gastrectomy)	Yes — vitamin B12 and iron (if deficient)
Mansfield et al.^b^, 2024 (USA) [36]	Surgical management of invasive gastric cancer (Expert opinion — UpToDate)	Gastric cancer patients	Yes - Vitamin B12, iron, vitamin D - Calcium ion and vitamin D (for bone health) - Bone density (especially for females)	Yes - B12 - Iron, calcium, and vitamin D (if deficient)
Mulder et al., 2016 (Canada) [39]	Report from the 17th Annual Western Canadian Gastrointestinal Cancer Consensus Conference; Edmonton, Alberta; 11–12 September 2015 (Consensus statement)	Gastric cancer patients	Yes — no details except suggestion of periodic assessment	Yes — including but not limited to vitamin B12 and iron (if deficient)
National Comprehensive Cancer Network^a,b^, 2024 (USA) [30]	NCCN Clinical Practice Guidelines in Oncology for Gastric Cancer (Version 1.2024—7/3/2024) (Guideline — reached consensus)	Gastric cancer patients	Yes - Including but not limited to vitamin B12, D, iron (CBC and iron levels), calcium, and zinc - Screening for osteopenia/osteoporosis screening for patients who are either 3-year post-surgery, over 50 years old, or post-menopausal	Yes - Routine supplementation of: vitamin B12, D, calcium, and a multivitamin and mineral complex (to include vitamins A, C, D, and E, folate, thiamine, copper, iron, magnesium, selenium, and zinc) - Vitamin B12 and iron (if deficient)
Rino et al., 2016 (Japan) [40]	Changes in fat-soluble vitamin levels after gastrectomy for gastric cancer (Narrative review)	Gastric cancer patients after gastrectomy	No	Yes — prophylactic vitamin supplementation *is not* recommended, only supplement if deficient
Rosania et al., 2016 (Germany) [41]	Nutrition in Patients with Gastric Cancer: An Update (Narrative review)	Gastric cancer patients	No	Yes — prophylactic vitamin B12 and iron
Shaw, 2014 (UK) [35]	Gastric cancer and nutrition (Expert opinion — book chapter)	Gastric cancer patient	Yes — B12 (for post partial gastrectomy)	Yes — B12 (parental supplementation post total gastrectomy; if deficient post partial gastrectomy)
Shitara et al., 2024 (Europe and Asia) [24]	Pan-Asian adapted ESMO Clinical Practice Guidelines for the diagnosis, treatment and follow-up of patients with gastric cancer (Guideline — reached consensus)	Asian patients with gastric cancer	Yes — no details	No
Teixeira Farinha et al.^a^, 2023 (France and Switzerland) [27]	Gastro-Intestinal Disorders and Micronutrient Deficiencies (Systematic review)	Oesophageal and gastric cancer patients	Yes — monitor vitamin levels (post partial gastrectomy)	Yes — in case of deficiency
Pancreatic cancer (***n*** = 7)				
Jacobson et al., 2023 (USA) [5]	Long‐term nutrition alterations after surgery for gastrointestinal cancers (Narrative review)	Patient with gastrointestinal cancers (including colorectal) after surgery (including Whipple procedure, gastrojejunostomies, and/or total gastrectomy)	No (post Whipple procedure)	Yes - Folate and iron (post Whipple procedure) - Vitamin C (optional; to enhance iron absorption)
Kluger et al., 2024 (USA) [45]	Total Pancreatectomy (Expert opinion — UpToDate)	Patients after total pancreatectomy	Yes — fat-soluble vitamins	Yes — when required to prevent development of conditions like osteoporosis
Mulliri et al.^a^, 2023 (France) [9]	Functional sequelae after pancreatic resection for cancer (Systematic review)	Pancreatic cancer patients after surgery	Yes - Sodium, potassium, phosphorus, calcium, magnesium (blood test) - Vitamins B12, A, D, and E, zinc, selenium, ferritin, and saturation coefficient of transferrin (assays)	Yes - Vitamin D, vitamin B12, and folinic acid - Iron, vitamins A, E, and K, phosphorus, and magnesium (if deficient) - Calcium (in the event of hypochlorhydria; calcium citrate is recommended)
Pappas et al., 2010 (USA) [42]	Nutrition and pancreaticoduodenectomy (Narrative review)	Patients after pancreaticoduodenectomy/Whipple procedure	Yes — suggested to have similar assessment as patients after Roux-en-Y gastric bypass patients	Yes — suggested to have similar supplementation as patients after Roux-en-Y gastric bypass patients
Petzel et al.^a,b^, 2017 (USA) [43]	Nutrition Implications for Long-Term Survivors of Pancreatic Cancer Surgery (Narrative review)	Patients after pancreatic surgery (applicable to patients with cancers of ampulla, distal common bile duct, or duodenum and others benign or precancerous growths)	Yes - Copper, zinc, selenium, magnesium, folate, vitamin B12 (methylmalonic acid), vitamin A (retinol binding protein), E (alpha-tocopherol), and 25-OH vitamin D - Bone mineral density	Yes — if deficient
Petzel et al.^a,b^, 2022 (USA) [44]	Nutrition in Pancreatic Cancer (Chapter 26) (Expert opinion — book chapter)	Long-term pancreatic cancer survivor	Yes — vitamin A, vitamin B6 (evaluate pyridoxal 5-phosphate, pyridoxic acid), vitamin B12 (evaluate CBC, vitamin B12, methylmalonic acid), vitamin D (evaluate 25-OH), vitamin E (evaluate alpha tocopherol), copper (evaluate copper ceruloplasmin), iron (evaluate CBC, ferritin, total iron binding capacity, iron), selenium, zinc	Yes — vitamins A, B6, B12, D, and E, copper, iron, selenium, zinc (if deficient)
Petzel et al.^a^, 2023 (USA) [46]	Physical Activity and Nutrition Optimization in Pancreatic Cancer (Chapter 9) (Expert opinion — book chapter)	Long-term survivors of pancreatic cancer	Yes - Monitor for micronutrient deficiency - Bone mineral density	Yes - In case of deficiency - Calcium citrate supplementation is recommended (in case of deficiency)

^a^Recommendations regarding the timing of surveillance not included in the current table, refer to Supplementary Table 2 for further details

^b^Recommendations regarding the dosage of supplementation not included in the current table, refer to Supplementary Table 2 for further details

### Gastric cancer

Nineteen publications with recommendations for surveillance of micronutrient deficiencies and/or supplementation for patients with gastric cancer after surgery were identified [5, 23, 24, 26–41]. These publications were led by authors or professional organisations from different countries (USA 6, Korea 2, Canada 2, Taiwan 1, UK 1, Germany 1, Japan 1, Europe 1, and mixed 3) (Table 3).

Publications consisted of eight reviews (6 narrative [5, 32–34, 40, 41], 1 systematic [27], and 1 meta-analysis [26]), five guidelines [23, 24, 28–30], four expert opinions (1 book chapter [35] and 3 UpToDate publications [36–38]), and two consensus statements [31, 39].

All except for three publications suggested monitoring of micronutrient status; 11 provided specific details on which vitamins and/or minerals to monitor (either post total or partial gastrectomy) [5, 28–37], with five lacking details [23, 24, 26, 27, 39]. Of these, seven (44%) specified the timing of surveillance (two were for ‘lifelong monitoring’), as outlined in Supplementary Table 2. Monitoring B12 status was most commonly recommended (total *n* = 10) [5, 28–33, 35–37], followed by iron (*n* = 8) [28, 30–34, 36, 37], calcium (*n* = 4) [28, 30, 34, 36], vitamin D (*n* = 3) [30, 31, 36], zinc (*n* = 2) [30, 31], copper (*n* = 1) [31], folate (*n* = 1) [34], and fat-soluble vitamins (*n* = 1) [34]. In addition, bone mineral density (or osteoporosis) testing was recommended in five publications [30–32, 34, 36].

There were 16 publications with recommendations for micronutrient supplementation, either as prophylaxis or in case of micronutrient deficiency [5, 26–32, 34–41]. Of these, 14 (88%) detailed which micronutrients to supplement, with only six including information on dosage (as outlined in Supplementary Table 2). The most common micronutrient supplement mentioned was vitamin B12 (*n* = 14; prophylactic supplementation was recommended in nine publications [5, 26, 28, 29, 31, 34, 36, 38, 41], supplementation in case of B12 deficiency in three publications [32, 37, 39], and two publications mentioned both instances [30, 35]). This was followed by recommendations for iron (*n* = 12) [5, 28–32, 34, 36–39, 41], calcium (*n* = 8) [26, 28–31, 34, 36, 38], vitamin D (*n* = 6) [26, 29–32, 36], folate (*n* = 3) [5, 31, 34], fat-soluble vitamins (*n* = 2) [34, 38], zinc (*n* = 1) [31], copper (*n* = 1) [31], and vitamin C (*n* = 1) [5] supplementation. Prophylactic multivitamin supplementation was recommended in three publications [30, 31, 34].

Among the practice guidelines and consensus statements (Table 2) from a group of experts, only four included detailed recommendations for micronutrient surveillance and/or supplementation, as outlined in Table 3 and Supplementary Table 2.

### Pancreatic cancer

As outlined in Table 3, seven publications discussed nutritional management in patients after pancreatic surgery [5, 9, 42–46], with two being surgery specific [42, 45] and two including patients with other resected cancers [5, 43]. These publication types were as follows: narrative reviews (*n* = 3) [5, 42, 43], systematic review (*n* = 1) [9], and expert opinions (*n* = 3, 2 book chapters [44, 46] and 1 UpToDate publication [45]). Six publications recommended monitoring pancreatic cancer survivors’ micronutrient status after surgeries, such as, but not limited to, pancreatectomy or pancreaticoduodenectomy [9, 42–46]. Four of the publications included specific recommendations for the timing of surveillance [9, 43, 44, 46], three of which also specified the type of micronutrients to be monitored [9, 43, 44] (see Supplementary Table 2).

Vitamin B12, vitamin A, vitamin D, vitamin E, selenium, and zinc were the most commonly mentioned micronutrients requiring monitoring (each in three publications [9, 43, 44]). Two of the publications recommended monitoring copper [43, 44], magnesium [9, 43], and iron (one explicitly outlined iron [44] and one recommended ferritin and saturated co-efficient of transferrin [9]) status. Other micronutrients mentioned included vitamin B6 [44], calcium [9], sodium [9], fat-soluble vitamins [45], folate [43], potassium [9], and phosphorus [9] (all once). Bone mineral density testing was recommended in two publications [43, 46].

All seven publications included recommendations for micronutrient supplementation: four in case of deficiency [43–46], one in both [9], and one prophylactically for patients after a Whipple’s procedure [5]. One narrative review suggested patients after pancreatic cancer surgery have supplementation as per recommendations for the patient cohort after Roux-en-Y gastric bypass or sleeve gastrectomy [42]. The following micronutrients were recommended to receive supplementation in the case of deficiencies: vitamin B12 (*n* = 2) [9, 44], iron (*n* = 2) [9, 44], vitamin A (*n* = 2) [9, 44], vitamin E (*n* = 2) [9, 44], vitamin D (*n* = 2) [9, 44], calcium (*n* = 2) [9, 46], vitamin K (*n* = 1) [9], vitamin B6 (*n* = 1) [44], copper (*n* = 1) [44], folinic acid (*n* = 1) [9], magnesium (*n* = 1) [9], phosphorus (*n* = 1) [9], selenium (*n* = 1) [44], and zinc (*n* = 1) [44]. Prophylactic supplementation of iron (*n* = 1), folate (*n* = 1), and vitamin C (*n* = 1) was recommended for patients after pancreaticoduodenectomy [5]. The suggestions provided by the included studies regarding vitamin and mineral supplementation and surveillance in UGI cancer survivors after surgery are summarised in Table 3.

## Discussion

To our knowledge, this is the first scoping review to explore and summarise recommendations from published guidelines and/or literature reviews on the surveillance and supplementation of micronutrients for long-term UGI cancer survivors after curative intent surgery. Among the 26 articles identified in this scoping review, only eight publications were from a group of experts using a documented systematic process to produce the recommendations [23–25, 28–31, 39]; seven documented reaching consensus among the experts involved [24, 25, 28–31, 39]. The majority of these guidelines were targeted at gastric cancer survivors, with one for oesophageal cancer and none for pancreatic cancer survivors. Many publications, including practice guidelines from established groups of experts, were excluded from the review due to not having nutrition-related information under surveillance or a cancer survivorship section [18, 19, 47–49], nutrition-related information specific to UGI cancer survivors [14, 16, 50], or any recommendations related to vitamin and mineral supplementation and surveillance of these micronutrient deficiencies [51]. The extensive list of publications excluded in this review highlights the lack of acknowledgement by the expert groups regarding the risk of micronutrient deficiencies among UGI cancer survivors during the survivorship phase, despite nutrition-related risk and symptoms affecting nutrient absorption, for patients after UGI cancer surgeries, being well known [5, 27, 34, 44, 52]. This is especially true for pancreatic and oesophageal cancer survivors given the current lack of recommendations.

The prevalence of micronutrient deficiencies among UGI cancer survivors is high. As previously discussed, nearly half of the 2627 gastric cancer survivors included in the meta-analysis had vitamin B12 deficiency after gastrectomy [7]. Patients after gastrectomy or oesophagectomy were found to have significantly lower vitamin D levels compared to the healthy population in another meta-analysis [26]. Other observational studies showed 65 to 78% of the cancer survivors had at least one micronutrient deficiency 6 months after oesophagectomy [6, 11, 53] and these micronutrient deficiencies could occur at a later stage [11]. The types of surgery and surgical techniques, including reconstruction methods, have been shown to be one of the factors affecting survivors’ nutritional status [42]. Whilst some functional modifications to the UGI tract may improve over time, the significant alteration of the tract following curative intent surgery is unlikely to completely recover. For example, the overall volume of (food) consumed at a time after total gastrectomy may improve over time but will not return to the same capacity as prior to surgery. Similarly, symptoms due to the rapid transit time of food in the GIT after oesophagectomy may improve with strategies and modification of eating behaviour, but require a lifelong adjustment. Other impacts such as loss of parietal cells in the stomach after total gastrectomy, bypassing the duodenum and jejunum, and pancreatic enzyme insufficiency after a Whipple procedure are unlikely to improve without supplementation and require long-term supplementation such as vitamin B12 and pancreatic enzyme replacement therapy [5, 35, 42, 54]. Given the potential risk of micronutrient deficiencies for cancer survivors after surgery, there is a need for long-term monitoring of the mineral and vitamin status of UGI cancer survivors, and some will benefit from prophylactic supplementation.

Whilst the current scoping review identified recommendations for the surveillance and supplementation of vitamins and minerals for UGI cancer survivors, recommendations for the type of micronutrient to monitor, timing of surveillance, and dosage of supplementation varied between publications. Within the recommendations available, there was either a lack of detail or an inconsistency between the recommendations. Whilst this discrepancy is to be expected — as current survivorship guidelines are largely consensus based — from a clinical perspective, this lack of consensus in monitoring and preventing micronutrient deficiencies affects the care for these long-term UGI cancer survivors [25, 30, 43, 55].

It is widely accepted that there is a lack of high-level clinical evidence available to inform guidelines. Whilst for the most part this is thought to stem from the changing nature of the survivorship paradigm, a contributing factor may be the low survival rates of these patients in the past and other competing survivorship-related issues such as symptom management and psychological factors (e.g. fear of cancer recurrence). However, with improvements in survival, it is important to obtain high-level evidence to inform guidelines that include recommendations for micronutrient surveillance and supplementation within the survivorship phase.

A strength of this study was it included an extensive search for relevant literature, including the use of six databases, 21 relevant websites, and a hand search. However, the publications included were limited to English texts only, which may have resulted in some relevant publications in other languages (e.g. German S3 guidelines [56]) being missed or excluded. Other study limitations include (1) publications before 2010 were not searched. Cancer survivorship is a relatively new concept with increasing attention [12] and improved survival in UGI cancer survivors with new treatment modalities and/or chemotherapy regimens [57, 58] since 2010. Excluding publications prior to 2010 is therefore unlikely to affect the outcome of the current scoping review, but we cannot be certain of this; (2) the selection criteria were highly specific. Some of the publications with general statements or discussions pertaining to the monitoring and supplementation of micronutrients were not presented as ‘recommendation statements’, rather they were excluded from the review [59]. For instance, ‘follow up should focus on nutrition support’ in ESMO practice guideline for oesophageal cancer [18] was deemed to not be micronutrient specific, and subsequently excluded from the review. The third study limitation was that the interpretation of the recommendations presented in the publications could be subjective. Many of the publications related to pancreatic cancer survivors were excluded due to the recommendations for surveillance of fat-soluble vitamin deficiency being in the context of fat malabsorption, as a result of pancreatic insufficiencies [54, 60, 61]. The study team did though have two reviewers to extract data independently and compare the results to ensure data interpretation was consistent with the published articles.

## Conclusion

In summary, this scoping review found limited recommendations to monitor micronutrient deficiencies for long-term UGI cancer survivors, especially oesophageal and pancreatic cancer survivors after surgery, with many of them being narrative reviews. Vitamin B12 was the most recommended vitamin to monitor and supplement for UGI cancer survivors, especially for gastric cancer survivors. This was followed by fat-soluble vitamin supplementation in pancreatic cancer survivors. The clinical implications would be regular monitoring of micronutrient status should be part of the long-term surveillance plan for UGI cancer survivors, and vitamin and supplementations should be considered despite limited details or lack of consensus in recommendations for the surveillance of micronutrient deficiencies (type, timing) and supplementation. Future research should focus on obtaining more robust evidence on nutritional status, including timing of checking micronutrient status of UGI cancer survivors and the type of mineral and vitamin supplementation. International evidence-based clinical guidelines on prevention and management of micronutrient deficiencies are needed to ensure consistent nutritional care to UGI cancer survivors.

## Supplementary Information

Below is the link to the electronic supplementary material.

Supplementary file1 (DOCX 3149 KB)
