# Supplementary material for: Recommendations on the surveillance and supplementation of vitamins and minerals for upper gastrointestinal cancer survivors: a scoping review
Source: J Cancer Surviv. 2024 Aug 29;20(2):506–21. doi: 10.1007/s11764-024-01666-4 (PMC12989006; doi:10.1007/s11764-024-01666-4)
Supplement: Supplementary file 1 — Supplementary file1 (DOCX 3149 KB) [file 11764_2024_1666_MOESM1_ESM.docx]

***Supplementary list 1:*** Medline Search Strategy

| 1 | Gastrointestinal Neoplasms/ or Gastrointestinal Diseases/ or Gastrointestinal Tract/ |
| --- | --- |
| 2 | ((upper gastrointestinal or UGI or gastrointestinal or digestive system) adj3 cancer*).mp. |
| 3 | ((upper gastrointestinal or UGI or gastrointestinal or digestive system) adj3 neoplasm*).mp. |
| 4 | ((?esophageal or stomach or gastric or small intestine or small bowel or pancrea*) adj3 cancer).tw. |
| 5 | (?esophagectomy or gastrectomy or pancreatectomy or cholecystectomy or Whipple* or pancreaticoduodenectomy).tw. |
| 6 | general surgery/ or surgical oncology/ |
| 7 | Digestive System Surgical Procedures/ |
| 8 | ((upper gastrointestinal or UGI or gastrointestinal or digestive system) adj3 (surger* or resection)).mp. |
| 9 | Cancer Survivors/ or Survivorship/ |
| 10 | (cancer adj3 survivor*).mp. |
| 11 | survivorship.mp. |
| 12 | (cure or cured or curative).mp. |
| 13 | Humans/ or human*.mp. |
| 14 | Adult/ or Middle Aged/ or (adult* or middle age*).mp. |
| 15 | or/1-4 |
| 16 | or/5-8 |
| 17 | or/9-14 |
| 18 | 15 and 16 and 17 |
| 19 | Outpatients/ or Postoperative Care/ or Aftercare/ or Postoperative Complications/ |
| 20 | (post adj3 (discharge* or operati* or surger*)).mp. |
| 21 | (outpatient* or communit*).mp. |
| 22 | (patient adj3 discharge*).mp. |
| 23 | after surgery.mp. |
| 24 | ((after or post) adj3 hospital*).mp. |
| 25 | (follow* up adj3 care*).mp. |
| 26 | or/19-25 |
| 27 | Dietary Supplements/ or Micronutrients/ |
| 28 | Nutrition Support/ or Nutrition Status/ |
| 29 | Vitamins/ or Minerals/ |
| 30 | (dietary supplement* or supplement*).mp. |
| 31 | (nutrition* support or nutrition* stat*).mp. |
| 32 | micronutrient.mp. |
| 33 | ((vitamin or mineral) adj3 supplement*).mp. |
| 34 | (vitamin B12 or thiamine or riboflavin or niacin or vitamin B6 or vitamin C or vitamin D or vitamin E or vitamin K).mp. |
| 35 | (folate or iron or calcium or iodine or magnesium or potassium or sodium or zinc).mp. |
| 36 | or/27-35 |
| 37 | Guideline/ or Practice Guideline/ or Reference Standards/ or Review/ or Systematic Review/ or Meta Analysis/ |
| 38 | ((cancer or oncology or nutrition*) adj3 (guideline* or recommendation* or standard*)).tw. |
| 39 | ((clinic* or best* or standard*) adj3 practice).tw. |
| 40 | (guideline* or recommend* or standard * or practice or consensus).tw. |
| 41 | (review or (systematic adj2 review) or meta analys*).tw. |
| 42 | 37 or 38 or 39 or 40 or 41 |
| 43 | 18 and 26 and 36 |
| 44 | 43 and 42 |
| 45 | limit 44 to (english language and yr="2021 -Current") |

**Supplementary Table 1: List of websites for government, organisation or professional body searched and date of accessed (n=21)**

| **Organisations Searched** | **Last Date Accessed** |
| --- | --- |
| **Professional organisations** | |
| European Society for Clinical Nutrition and Metabolism (ESPEN) | 16/04/24 |
| American Society for Parental and Enteral Nutrition (ASPEN) | 16/04/24 |
| Australasian Society for Parental and Enteral Nutrition (AuSPEN) | 16/04/24 |
| European Society for Medical Oncology (ESMO) | 27/03/24 |
| American Society of Clinical Oncology (ASCO) | 01/04/24 |
| Clinical Oncology Society of Australia (COSA) | 07/04/24 |
| Practice-Based Evidence in Nutrition | 10/04/24 |
| British Society of Gastroenterology | 28/03/24 |
| National Institute for Health and Care Excellence (NICE) | 01/04/24 |
| International Society of Geriatric Oncology (SIOG) | 01/04/24 |
| European Society of Gastrointestinal Endoscopy (ESGE) | 01/04/24 |
| Pancreatic Cancer Action Network (PanCAN) | 10/04/24 |
| National Comprehensive Cancer Network (NCCN) | 02/04/24 |
| UpToDate | 07/04/24 |
| International Study Group on Pancreatic Surgery (ISGPS)* | 01/04/24 |
| **Cancer Organisations** | |
| European Organisation for the Research and Treatment of Cancer (EORTC) | 07/04/24 |
| World Cancer Research Fund (WCRF) | 02/04/24 |
| Cancer Council Australia | 01/04/24 |
| American Cancer Society (ACS) | 01/04/24 |
| **Government Sources** | |
| Cancer Australia | 07/04/24 |
| National Cancer Institute | 10/04/24 |

*** Attempted to search but no website available**

**Supplementary** Table 2: Guidelines/recommendations for timing of micronutrient surveillance and dosage of supplementation in upper gastrointestinal tract cancer survivors post curative intent treatment (n=15).

| ***First author, year***  ***(country)*** | ***Title***  ***(publication type)*** | ***Detailed recommendations for***  ***surveillance of micronutrient status***  ***(timing)*** | ***Detailed recommendations for***  ***micronutrient supplementation***  ***(dosage and timing if available)*** |
| --- | --- | --- | --- |
| **Oesophageal Cancer (n=2)** | | | |
| National Comprehensive Cancer Network, 2024  (USA)  [25] | NCCN Clinical Practice Guidelines in Oncology for Esophageal and Esophagogastric Junction Cancers (Version 1.2024)  (Guideline – reached consensus) | First six months post surgery, monitor **calcium**, **folic acid**, **B vitamins** and **vitamin D** (in the context of monitoring for malnutrition) |  |
| Teixeira Farinha et al, 2023  (France & Switzerland)  [27] | Gastro-Intestinal Disorders and Micronutrient Deficiencies  (Systematic review) | Monitor vitamin levels (at 1 month, 3 months, 6 months, 1 year, 2 years and up to 5 years post surgery) |  |
| **Gastric Cancer (n=9)** | | | |
| Cobani et al,  2023  (USA)  [32] | Gastric Cancer Survivorship: Multidisciplinary Management, Best Practices and Opportunities  (Narrative Review) | **B12** (if deficient; every 3 months for up to 3 years, then every 6 months for up to 5 years, then annually)  **Iron** (if deficient; annually, at least) | **Vitamin D** (if at risk for osteoporosis; 16,000 IU of vitamin D3) |
| DeManzoni, et al 2016  (Italy)  [28] | The Italian Research Group for Gastric Cancer (GIRCG) guidelines for gastric cancer staging and treatment 2015  (Guideline – reached consensus) | Lifelong monitoring |  |
| Hsu et al, 2019  (Taiwan)  [31] | Taiwan nutritional consensus on the nutrition management for gastric cancer patients receiving gastrectomy  (Consensus statement) | **B12** (post subtotal gastrectomy; every 6 months)  **Iron** {serum ferritin & haemoglobin} (eg. every 6 months)  **Bone mineral density** (eg. annually)  **25-hydroxy vitamin D** (eg. annually) | **B12**   - Post total gastrectomy: prophylactic intramuscular, subcutaneous or oral – eg. 1000mcg intramuscular in alternate months - Post subtotal gastrectomy: mild deficiency = oral supplementation of 500-1000mcg / day; severe deficiency = intramuscular or subcutaneous of 1000-2000mcg / month   **Iron** (if deficient, example dosage of 150-300 of elemental iron daily, for 4-6 months)  **Folate** (if deficient; 5mg daily)  Prophylactic daily **multivitamin** supplement (eg. multivitamin containing 250mg of calcium and 400IU of vitamin D) |
| Kim et al, 2023  (Korea)  [33] | Advances, breakthroughs, and challenges in gastric cancer surgery  (Narrative Review) | Lifelong monitoring |  |
| Kim et al, 2023  (Korea)  [29] | Korean Practice Guidelines for Gastric Cancer 2022: An Evidence-based, Multidisciplinary Approach  (Guidelines) |  | **B12** (intramuscular or high oral dosage {1,500mcg daily}) |
| Malik et al,  2020  (USA)  [34] | Nutritional Implications in Preparing Patients for Total Gastrectomy  (Narrative Review) | Bone mineral density (every 1-2 years post total gastrectomy) | **B12** (oral supplementation of 1-2mg daily or intramuscular/subcutaneous supplementation of 1mg every month, lifelong)  **Folate** (if deficient; 5mg daily)  **Iron** (if deficient; 150mg of iron polysaccharide or 67mg of elemental iron 1-2 times daily)  **Calcium** (patients with known bone disease; 1200mg daily, or equivalent)  **Vitamin D** (if deficient; 50,000 IU for 8-12 weeks followed by maintenance dosage)  *Recommendations are post total gastrectomy |
| Mansfield et al,  2024  (USA)  [36] | Surgical management of invasive gastric cancer  (Expert Opinion - UpToDate) |  | **B12** (intramuscular once monthly OR daily sublingual B-complex) |
| National Comprehensive Cancer Network, 2024  (USA)  [30] | NCCN Clinical Practice Guidelines in Oncology for Gastric Cancer (Version 1.2024 - 7/3/2024)  (Guideline – reached consensus) | **Vitamin B12** (lifelong monitoring; at least every 6 months if the patient is not supplementing B12 parenterally)  **Iron** (lifelong monitoring; CBC and iron levels annually, at a minimum)  **Calcium & Zinc** (lifelong monitoring)  **Vitamin D** (lifelong monitoring following local practice)  **Osteopenia/osteoporosis screening** (3 years post gastrectomy)   - Manage low bone density as per pre-established national guidelines. | If deficient supplement:   - **B12** (as per local practice) - **Iron** (as per local practice; when possible, avoid enteric coated and / or sustained release iron formulations, and instead supplement iron orally or intravenously)   Routine supplementation of:   - **Vitamin D** (as per local practice) |
| Teixeira Farinha et al  2023  (France & Switzerland)  [27] | Gastro-Intestinal Disorders and Micronutrient Deficiencies  (Systematic review) | Post partial gastrectomy monitor vitamin levels (at 1 month, 3 months, 6 months, 1 year, 2 years and up to 5 years post-surgery) |  |
| **Pancreatic Cancer (n=4)** | | | |
| Mulliri et al,  2023  (France)  [9] | Functional sequelae after pancreatic resection for cancer  (Systematic review) | **Sodium**, **potassium**, **phosphorus**, **calcium, magnesium**   - Monitor at M0, M1, M6, M12, M18, M24, and then yearly thereafter.   **Vitamin A, D, E, B12**, **ferritin**, **saturation coefficient of transferrin**, **zinc**, **selenium**   - Monitor assays at M0, M6, M12, M24, and then yearly thereafter. |  |
| Petzel et al,  2017  (USA)  [43] | Nutrition Implications for Long-Term Survivors of Pancreatic Cancer Surgery  (Narrative Review) | **Bone mineral density**   - Baseline evaluation within 2 years post surgery.   - If normal: follow up every 5 years   - If abnormal: follow up annually)   **Copper**, **zinc**, **selenium**, **folate**, **vitamin B12**, methylmalonic acid, **Vitamin A** (retinol binding protein), **Vitamin E** (alpha-tocopherol) , **25-OH Vitamin D**, **Magnesium**,   - Baseline evaluation within 1 year post surgery or sooner if patient develops clinical signs / symptoms of deficiency / malabsorption.   - If normal: follow up annually   - If abnormal: attempt to replete levels, and recheck after 3 months | **B12** (if deficient; intramuscular monthly supplementation of 1000 ug of cyanocobalamin) |
| Petzel et al  2022  (USA)  [44] | Nutrition in Pancreatic Cancer (Chapter 26)  (Expert Opinion – Book Chapter) | Within 1 year post surgery or sooner if patient develops clinical signs / symptoms of deficiency / malabsorption; if “normal” follow up annually and if deficient supplement and recked 3 months later:   - **Vitamin A** - **Vitamin B6** (evaluate pyridoxal 5-phosphate {PLP}, pyridoxic acid) - **Vitamin B12** (evaluate CBC, Vitamin B12, methylmalonic acid) - **Vitamin D** (evaluate 25-OH) - **Vitamin E** (evaluate alpha tocopherol) - **Copper** (evaluate copper ceruloplasmin) - **Iron** (evaluate CBC, ferritin, total iron binding capacity, iron) - **Selenium** - **Zinc** | If deficient:   - **Vitamin A** (30,000 IU retinol palmitate, 1 daily for 4 weeks) - **Vitamin B6** (60mg pyridoxine daily for 3 weeks) - **Vitamin B12** (1000 mcg cyanocobalamin intramuscular injectional, once a month) - **Vitamin D** (50,000 IU of cholecalciferol or ergocalciferol, once a week for 8 weeks OR 6000 IU of cholecalciferol or ergocalciferol once a day for 8 weeks) ** - **Vitamin E** (400 IU once daily for 2 weeks) - **Copper** (3–8 mg elemental copper, per day until levels normalise) - **Iron** (150-200 elemental iron daily, or every other day in 2 or 3 divided doses) - **Selenium** (100 mcg selenium, once a day until levels normalise) - **Zinc** (50-60mg elemental zinc, 1-2 times a day for 3 months)   For maintenance:   - **Vitamin B12** (1000 mcg cyanocobalamin intramuscular injectional once a month OR 1000 mcg vitamin B12, once daily) - **Vitamin D** (50,000 IU of cholecalciferol or ergocalciferol once every other week OR 1500 - 2000 IU of cholecalciferol, once a day) *   * NOTE: patients with obesity or patients who have malabsorption issues, may require a different dosage; 3000–6000 IU cholecalciferol, once a day  ** NOTE: patients with obesity or patients who have malabsorption issues, may require a different dosage; 50,000 IU of cholecalciferol or ergocalciferol., 3 times a week for 4–8 weeks OR 6000–20,000 IU cholecalciferol or ergocalciferol, once a day for 8 weeks |
| Petzel, 2023  (USA)  [46] | Physical Activity and Nutrition Optimization in Pancreatic Cancer (Chapter 9)  (Expert Opinion) | Within 1 year (or sooner if the patient develops signs/symptoms of deficiency or malabsorption)   - If serum levels are “normal” follow up annually - If deficient, supplement and recheck 3 months later |  |
